# Supplementary material for: TRPV3-ANO1 interaction positively regulates wound healing in keratinocytes
Source: Commun Biol. 2023 Jan 23;6:88. doi: 10.1038/s42003-023-04482-1 (PMC9870996; doi:10.1038/s42003-023-04482-1)

Supplementary Figure 1.

Characteristics of the currents in the presence of high intracellular calcium in NHEK

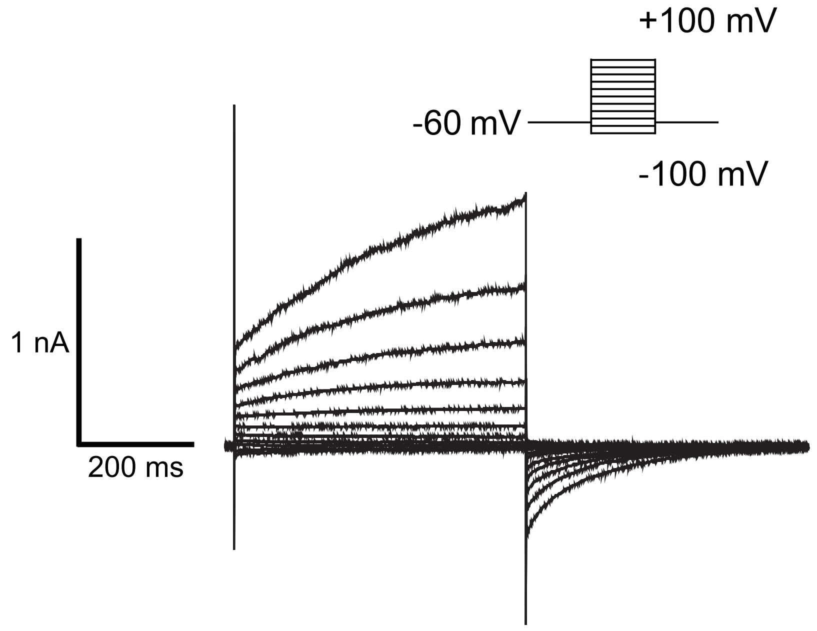

Representative trace of NHEK is shown. Step pulses for 500 msec were applied between -100 mV and +100 mV with 20 mV increments from -60 mV. NMDG-Cl bath solution and pipette solutions were used. Free calcium in the pipette solution was 500 nM.

Supplementary Figure 2. TRP channels expression in NHEKs

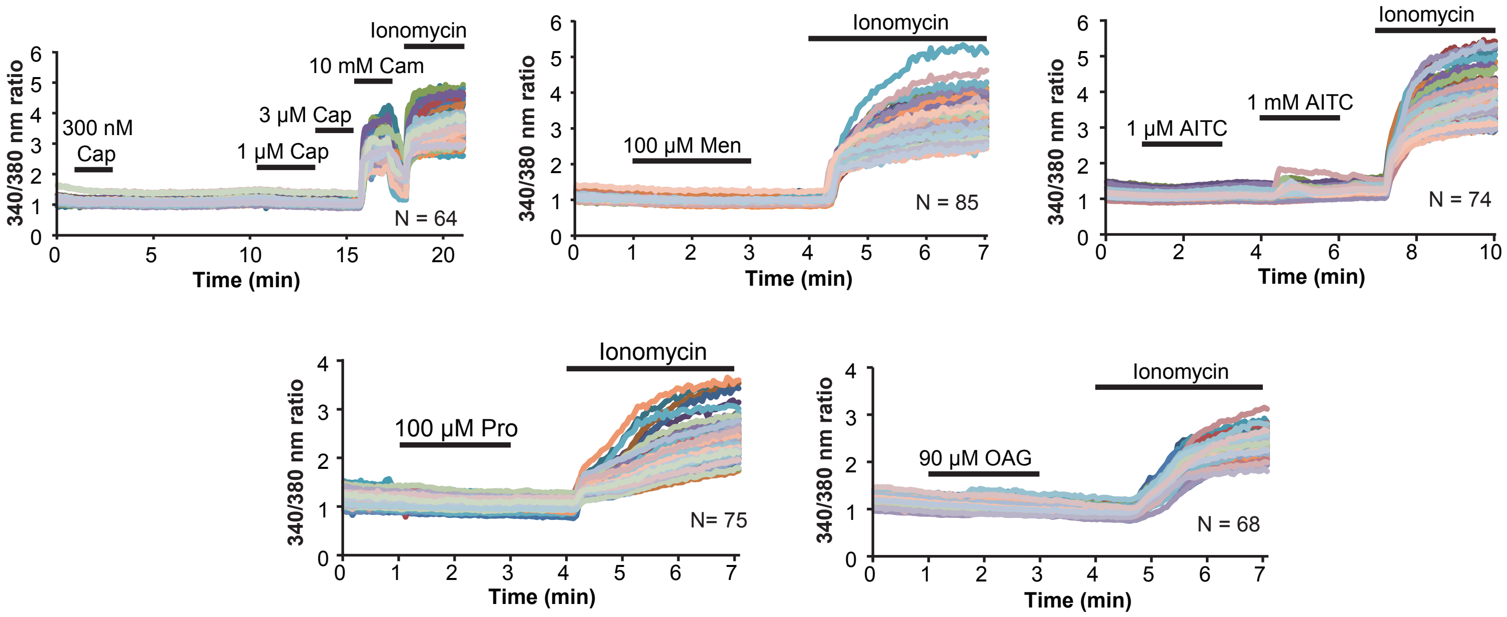

Calcium imaging in NHEKs is illustrated. Cam: Camphor, a TRPV3 agonist; GSK: GSK1016790A, a TRPV4 agonist; Cap: Capsaicin, a TRPV1 agonist; Men: Menthol, a TRPM8 agonist; AITC: Allyl isothiocyanate, a TRPA1 agonist; Pro: Probenecid, a TRPV2 agonist; OAG: 1-oleoyl acetyl-sn-glycerol, a TRPC6 agonist.

Supplementary Figure 3. No contribution of TRPV4 to camphor-induced currents

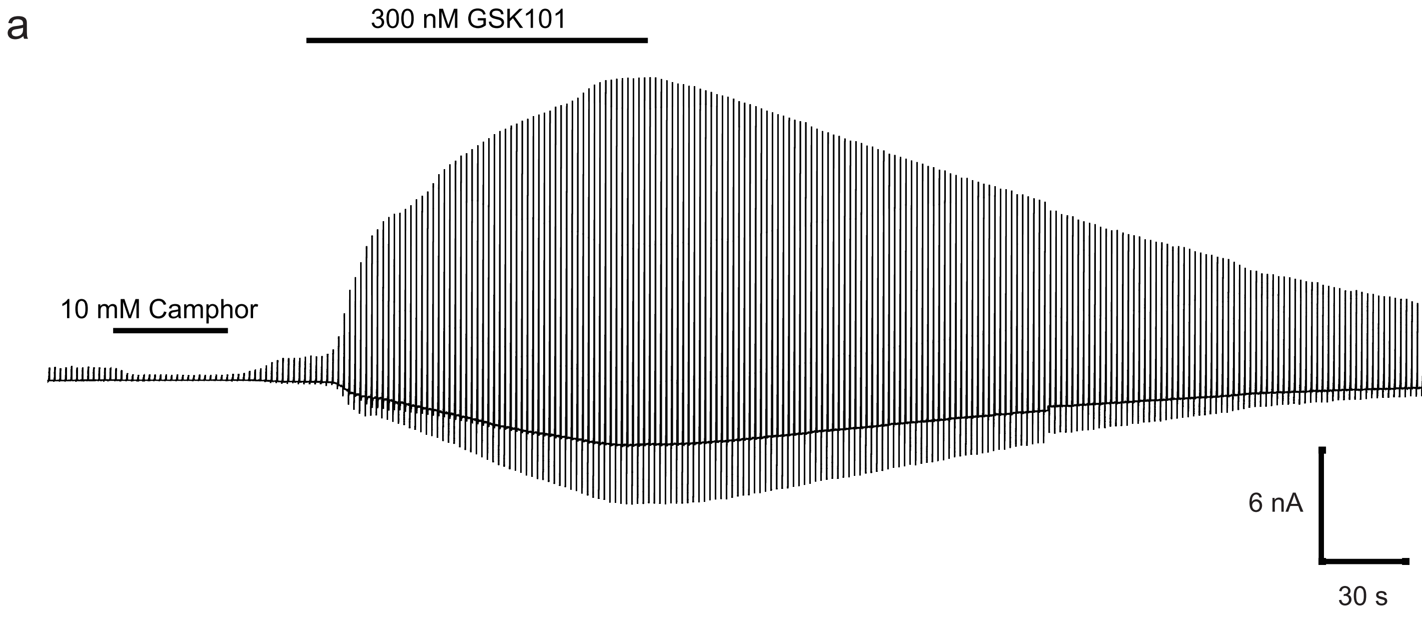

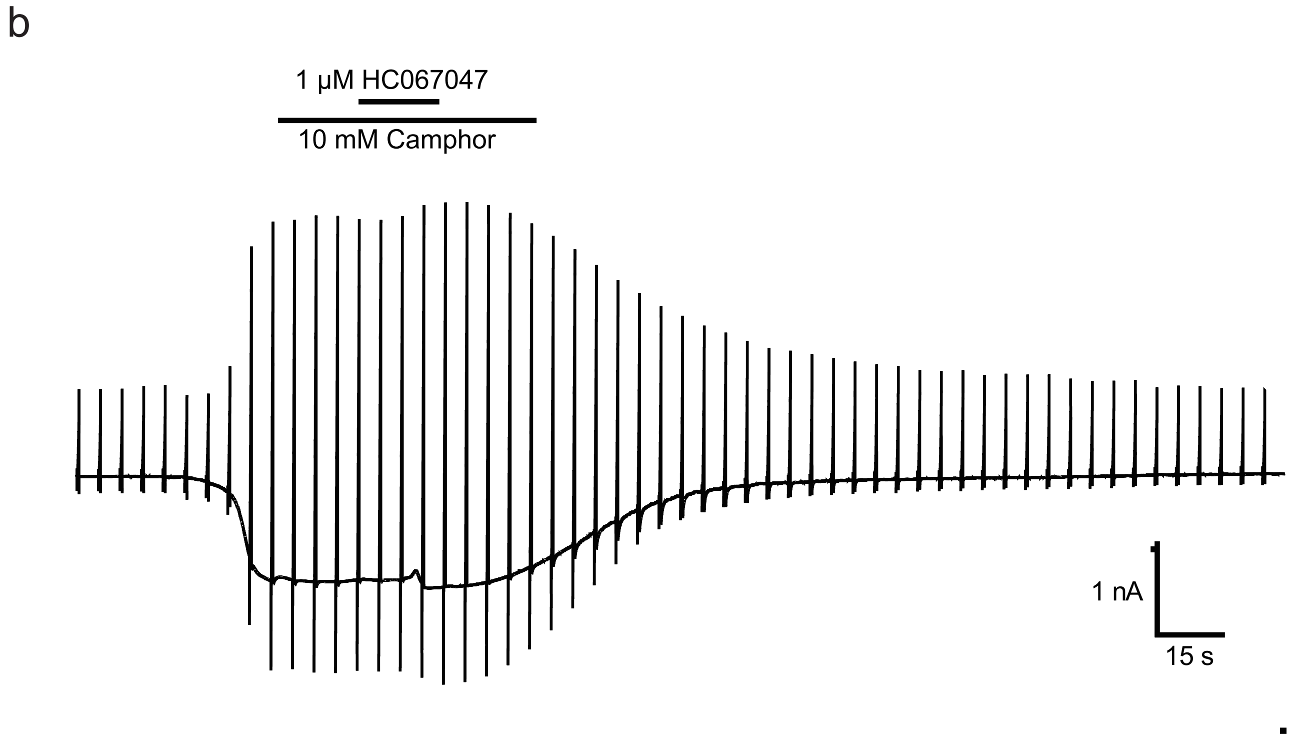

(a) Representative trace of HEK293T cells expressing hTRPV4. Camphor (10 mM) did not induce currents in HEK293T cells expressing hTRPV4. Data is collected using a NaCl bath solution containing 2 mM Ca^{2+} and KCl pipette solution. Holding potential was -60 mV and ramp-pulses were applied from -100 to +100 mV for 300 ms duration every 5 sec.

(b) Representative trace of the camphor-induced currents with a TRPV4 inhibitor, HC067047, using an NMDG-Cl bath solution in NHEK. The pipette solution contained 140 mM NMDG-Cl and 100 nM free calcium. The holding potential was -60 mV and ramp-pulses were applied from -100 to +100 mV for 300 ms duration every 5 sec.

Supplementary Figure 4.

Effect of a TRPV3 inhibitor, dyclonine, on camphor-induced currents

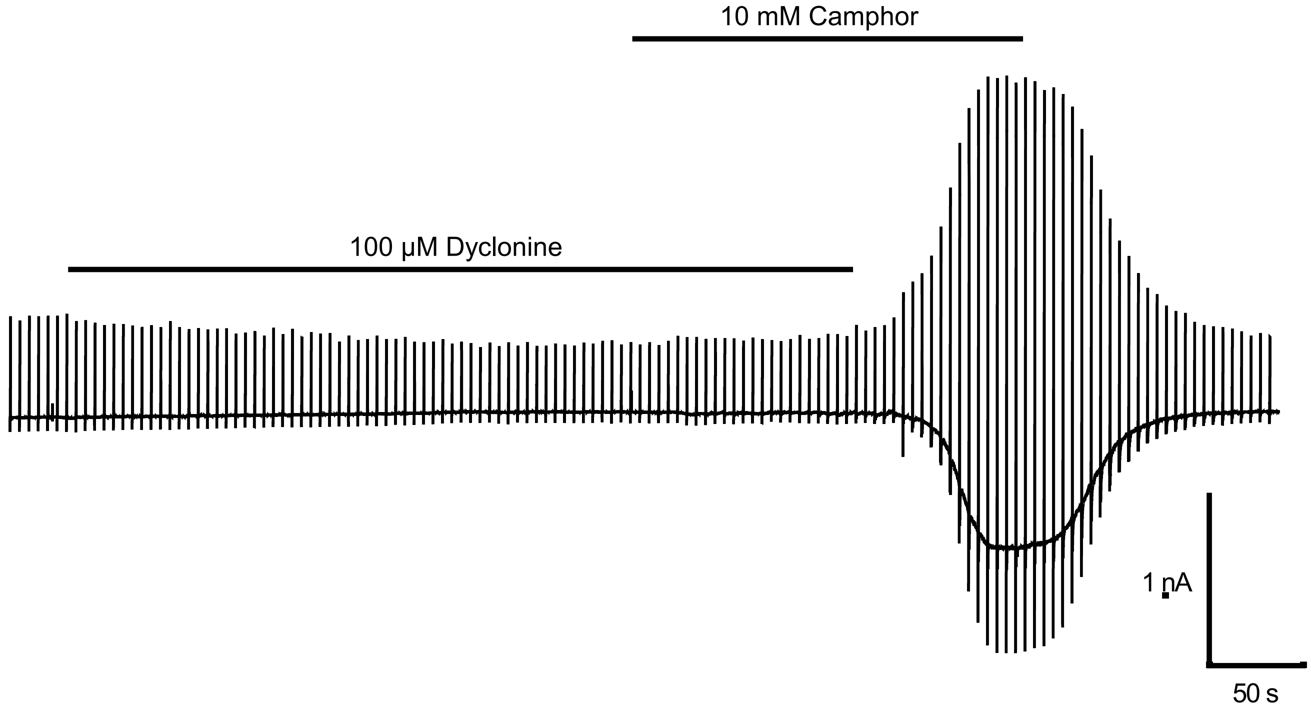

Camphor-induced current with a TRPV3 inhibitor, dyclonine, using an NMDG-Cl bath solution in NHEK. The pipette solution contained 140 mM NMDG-Cl and 100 nM free calcium. The holding potential was -60 mV and ramp-pulses were applied from -100 to +100 mV for 300 ms duration every 5 sec.

Supplementary Figure 5.

Characteristics of the camphor-induced currents in NHEK

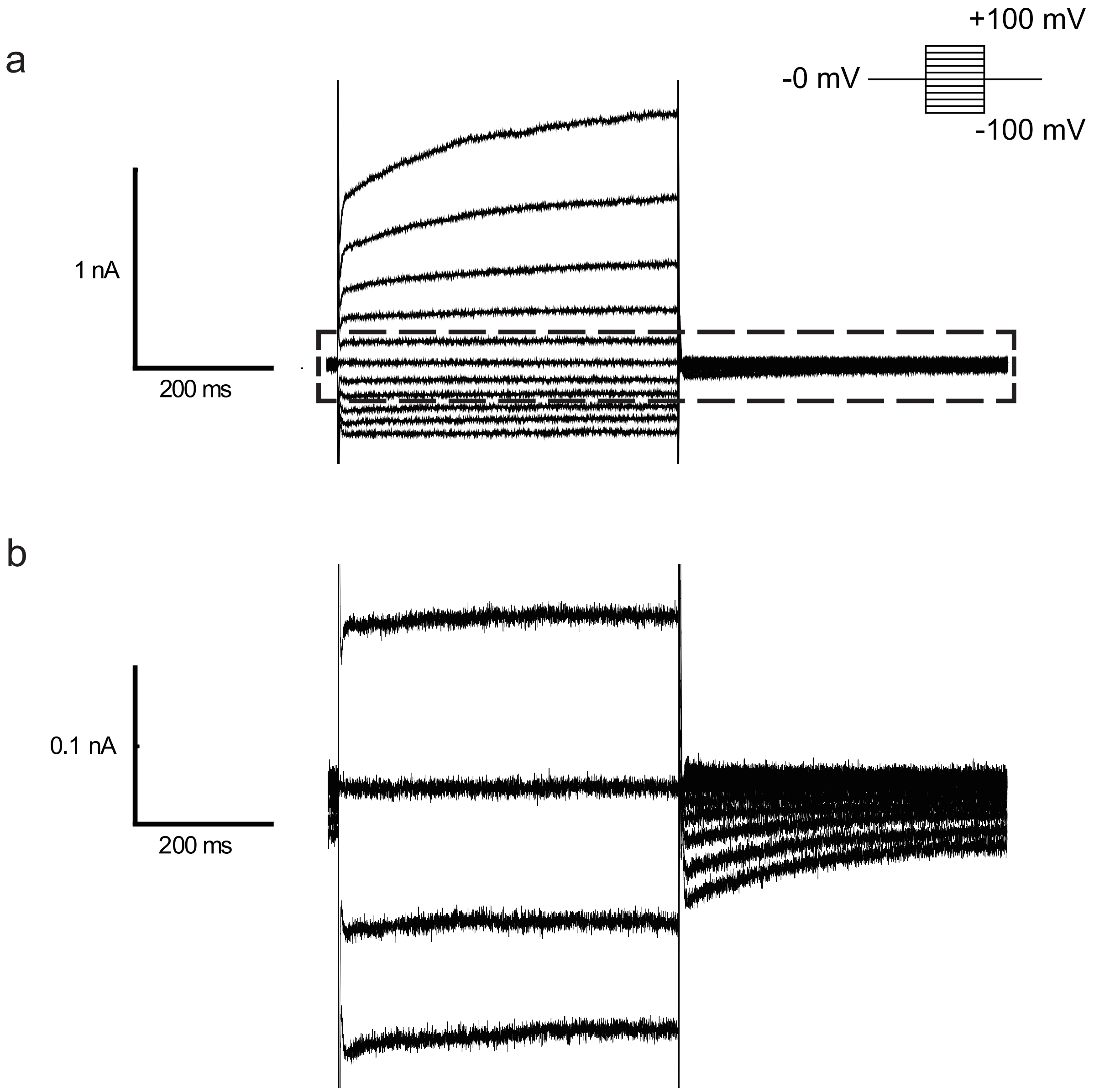

(a) Representative trace of NHEK is shown. Step pulses for 500 msec were applied between -100 mV and +100 mV with 20 mV increments from 0 mV. NMDG-Cl bath solution and pipette solutions were used. Free calcium in the pipette solution was 100 nM.

(b) Magnification of the dotted square in (a).

Supplementary Figure 6.

Effect of a TRPV3 inhibitor on cell migration/proliferation in theculture inserts

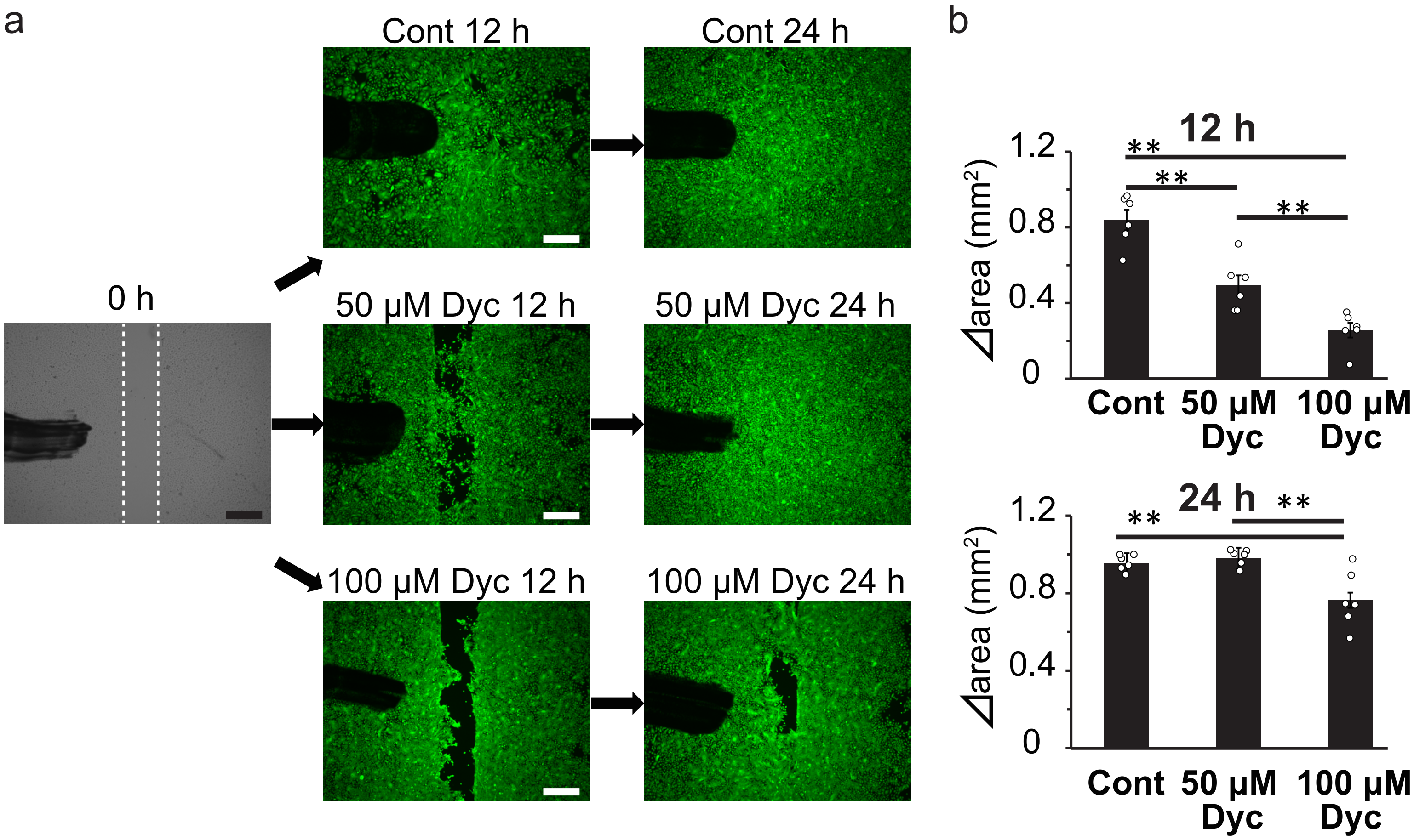

(a) Culture insert assay in media with or without a TRPV3 inhibitor, dyclonine (Dyc). Bright field at 0 h and calcein staining at 12 and 24 h. White dotted lines at 0 h indicate borders of the cells. Scale bars indicate 500 μm.

(b) Measurements of increased areas (⊿ area) at 12 h or 24 h in the medium with or without Dyc.

Data represent means ± S.E.M (N = 6).

Statistical significance was determined with Bonferroni correction. **, $p < 0.01$.

Supplementary Figure 7.

Effect of a TRPV4 inhibitor on cell migration/proliferation in the culture insert assay

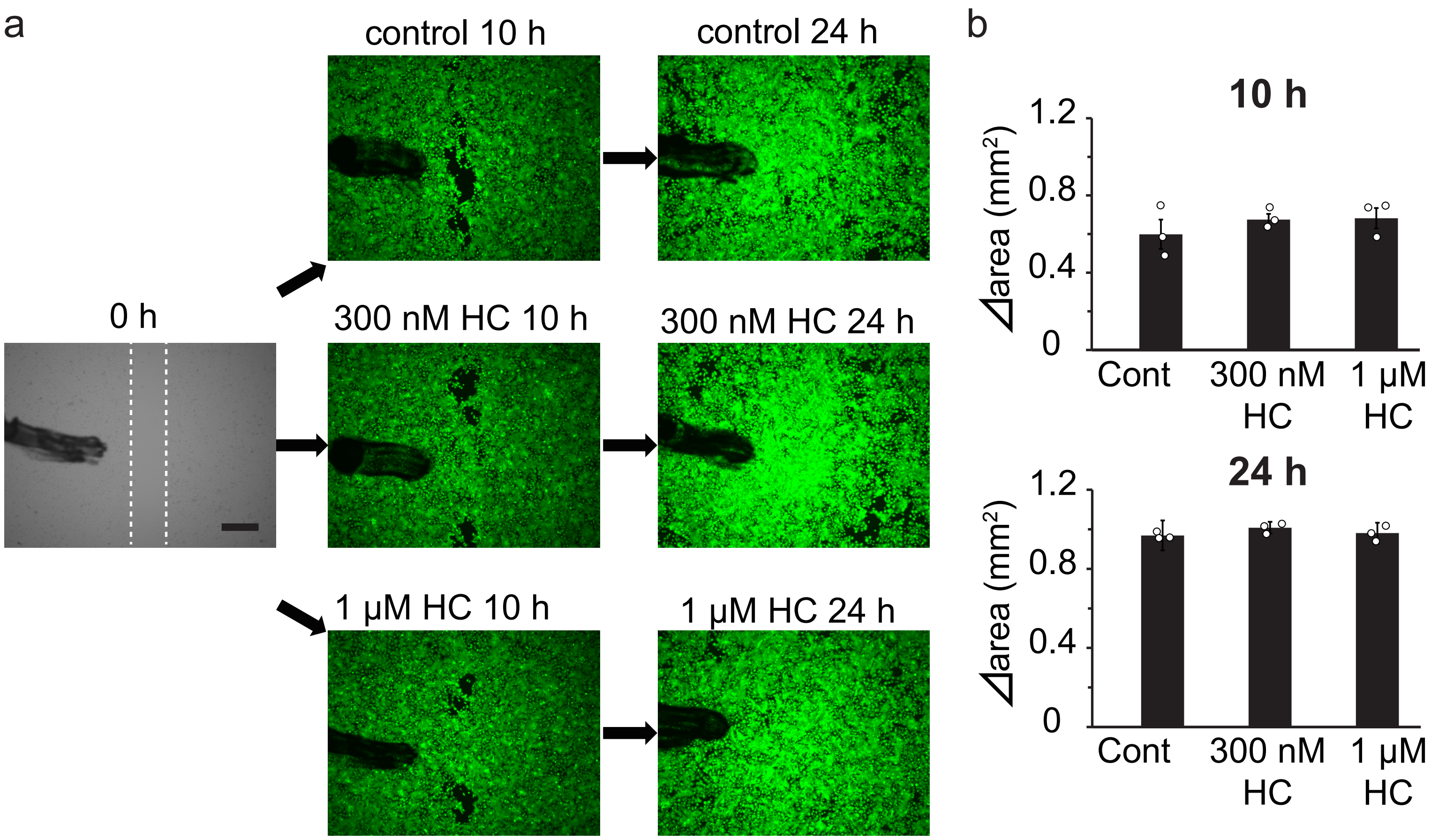

(A) Culture insert assay in media with or without a TRPV4 inhibitor, HC067047 (HC). Bright field at 0 h and calcein staining at 10 and 24 h. White dotted lines at 0 h indicate borders of the cells. Scale bars indicate 500 μm.

(B) Measurements of increased areas (⊿ area) at 10 h or 24 h in the medium with or without HC. Data represent means ± S.E.M (N = 3). Statistical significance was determined with Bonferroni correction. No significant differences were found.

Supplementary Figure 8.

Effects of an ANO1 inhibitor on ANO1 protein levels

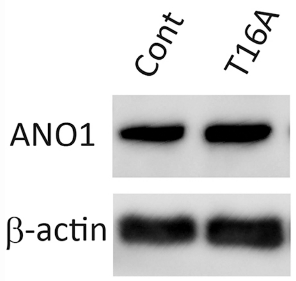

representative images of Western blotting for ANO1

Supplementary Figure 9.

Effect of another ANO1 inhibitor on cell migration/proliferation in the culture insert assay

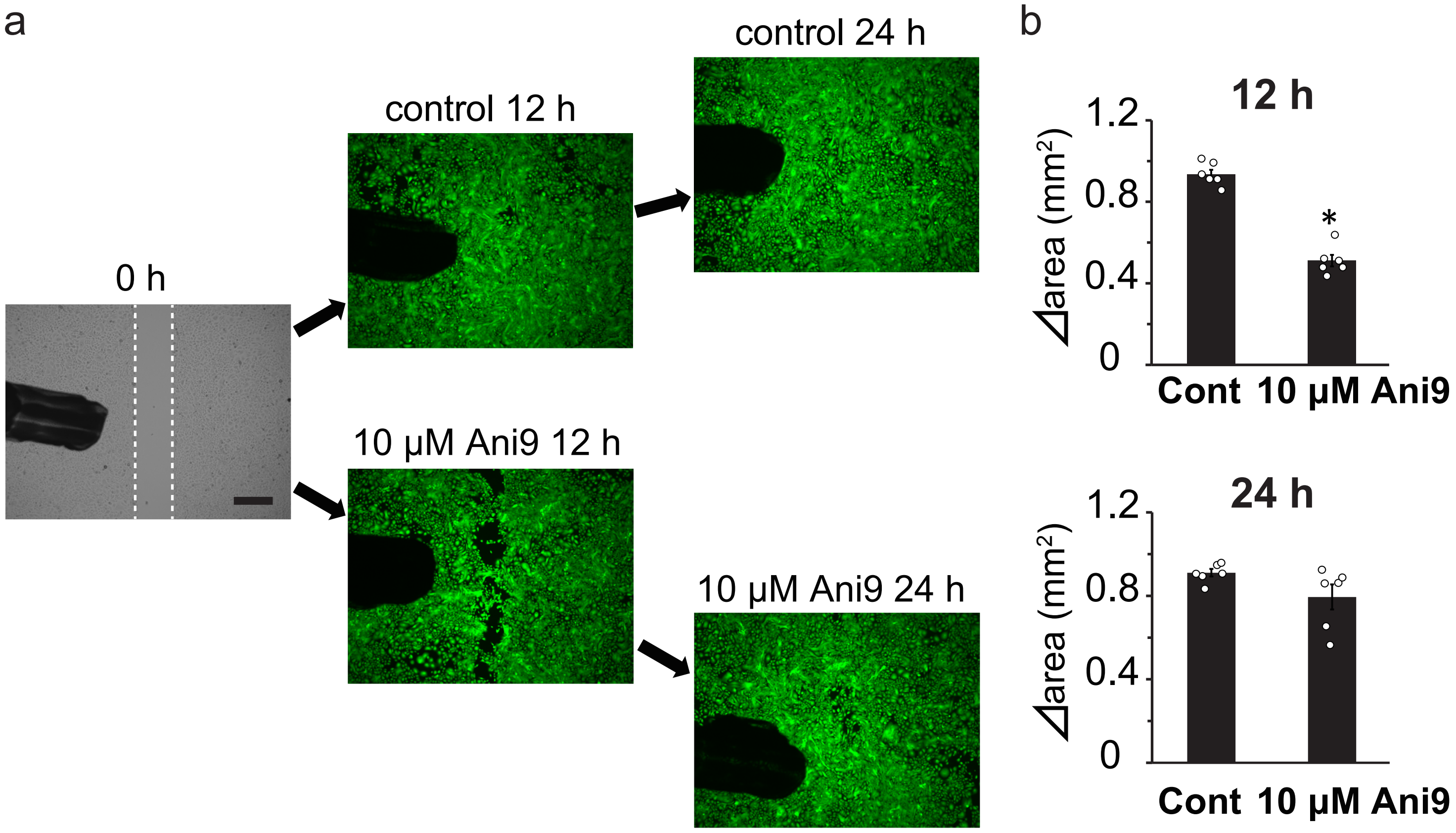

(a) Culture insert assay in medium with or without an ANO1 inhibitor, Ani9. Bright field at 0 h and calcein staining at 24 h. White dotted lines at 0 h indicate borders of the cells. Scale bars indicate 500 μm.

(b) Measurements of increased areas (⊿ area) at 12 h or 24 h in the medium with or without Ani9. Data represent means ± S.E.M (N = 6). Statistical significance was determined with Student's t-test. *, $p < 0.05$

Supplementary Figure 10.

Effects of an ANO1 inhibitor on NHEK differentiation

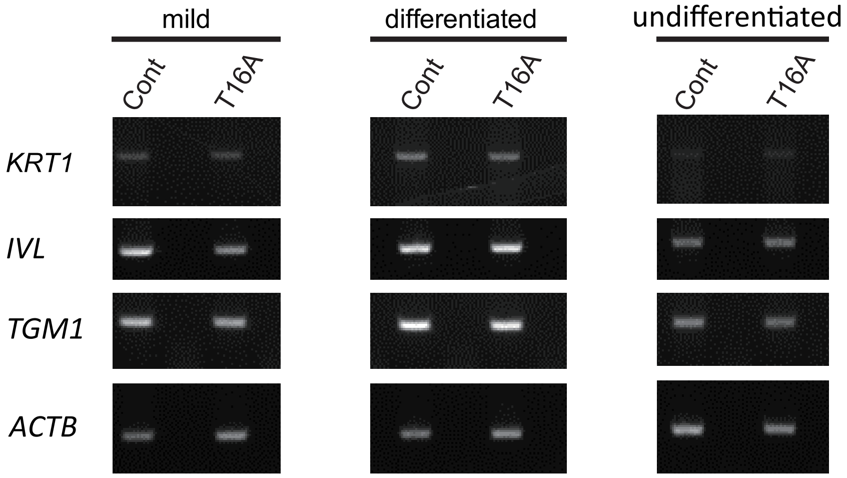

RT-PCR of differentiation marker genes in NHEKs

KRT1: keratin 1, *IVL*: involucrin, *TGM1*: transglutaminase 1, *ACTB*: β-actin

Differentiation was induced by the following conditions.

Mild: cultured at medium cell density in 0.15 mM calcium medium for 2 days.

Differentiated: cultured at high cell density in 1.5 mM calcium medium for 3 days.

Undifferentiated: cultured at low cell density in 0.15 mM calcium medium for 3 days.

Treatment with 10 μM T16A was conducted for 13 h.

Supplementary Figure 11 (cropped blots to the Figures)

Fig.1a

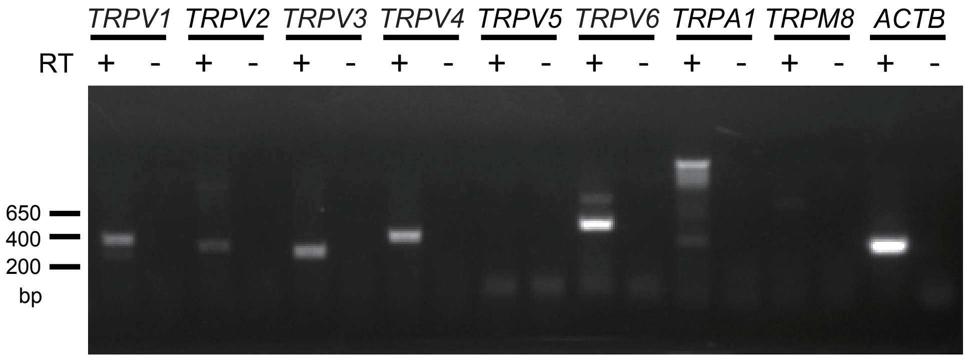

Fig.1b

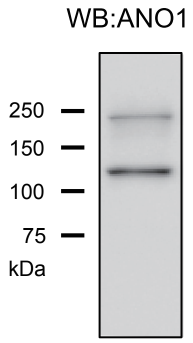

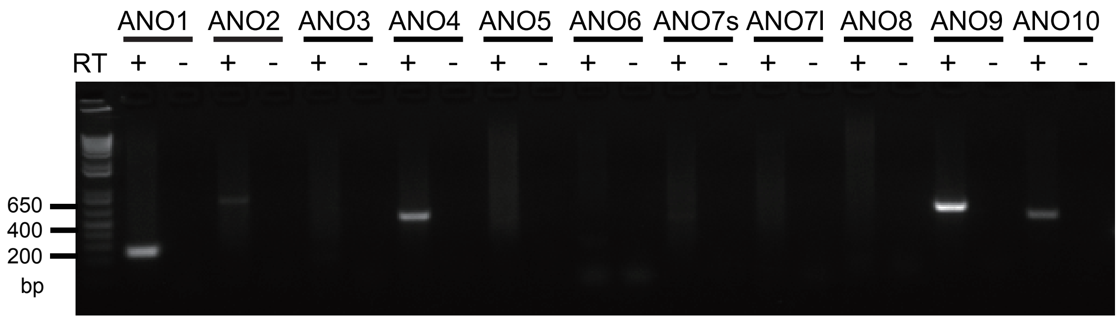

Fig.2c

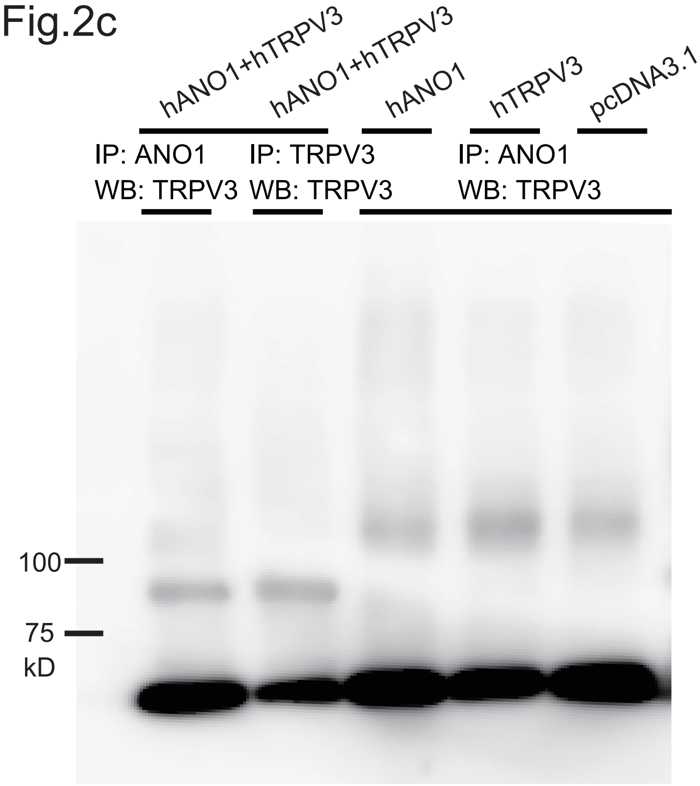
hANO1+hTRPV3
hANO1+hTRPV3
hANO1
hTRPV3
pcDNA3.1
IP: ANO1
WB: TRPV3
IP: TRPV3
WB: TRPV3
IP: ANO1
WB: TRPV3
100
75
kD

Fig.7a

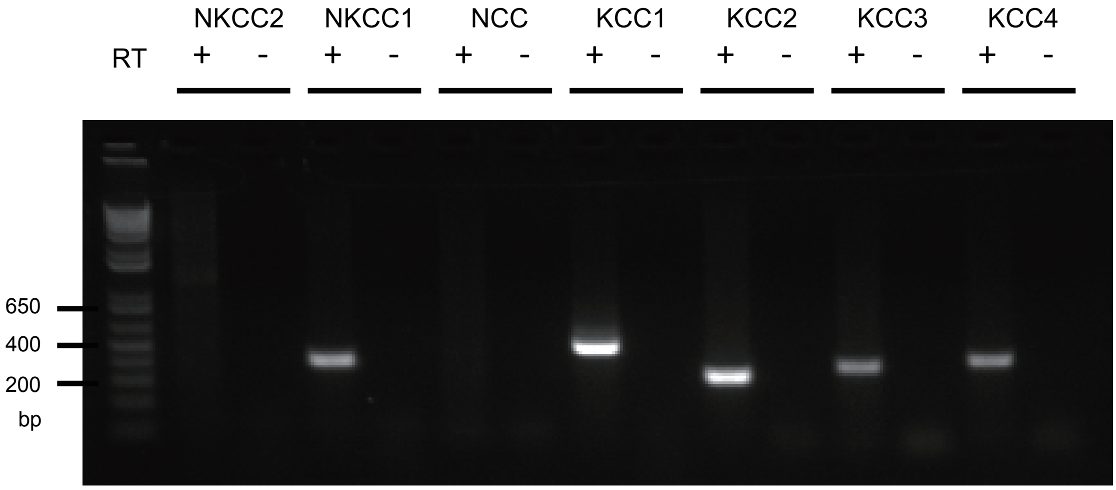

Fig.8b

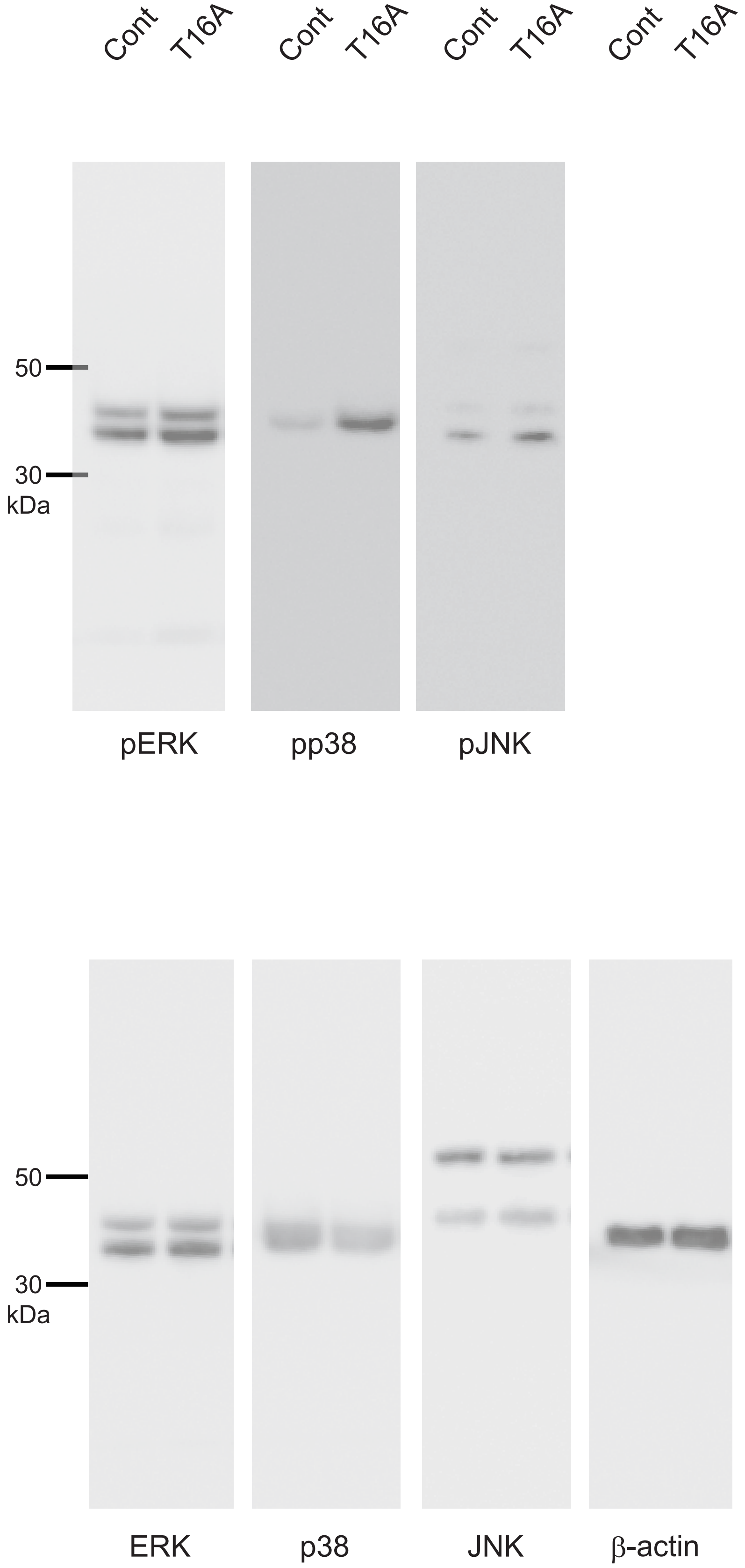
Cont
T16A
Cont
T16A
Cont
T16A
Cont
T16A
50
30
kDa
pERK
pp38
pJNK
50
30
kDa
ERK
p38
JNK
β-actin

Supplementary Figure 8

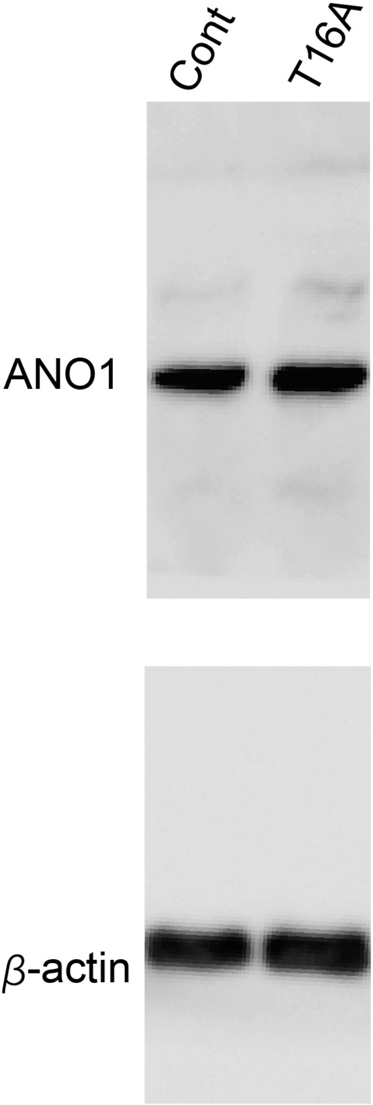

Supplementary Figure 10.

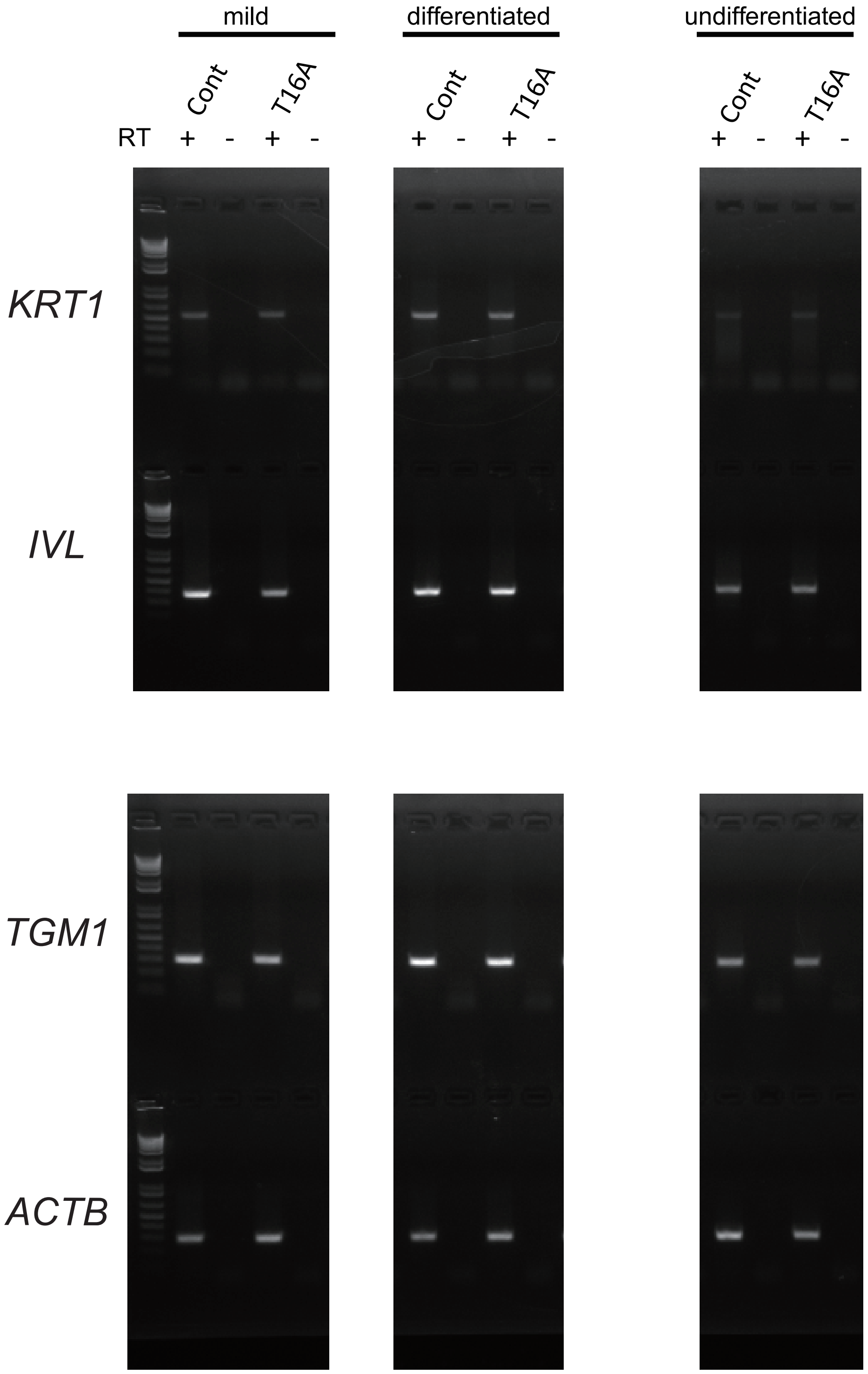

Supplement: Supplementary file 2 — Supplementary Information [file 42003_2023_4482_MOESM2_ESM.pdf]
