# Supplementary material for: TRPV3-ANO1 interaction positively regulates wound healing in keratinocytes
Source: Commun Biol. 2023 Jan 23;6:88. doi: 10.1038/s42003-023-04482-1 (PMC9870996; doi:10.1038/s42003-023-04482-1)
Supplement: Supplementary file 5 — Reporting Summary [file 42003_2023_4482_MOESM5_ESM.pdf]

nature research

Corresponding author(s): Makoto Tominaga

Last updated by author(s): Dec 28, 2022

Reporting Summary

Nature Research wishes to improve the reproducibility of the work that we publish. This form provides structure for consistency and transparency in reporting. For further information on Nature Research policies, see our Editorial Policies and the Editorial Policy Checklist.

Statistics

For all statistical analyses, confirm that the following items are present in the figure legend, table legend, main text, or Methods section.

n/a	Confirmed	
☐	☒	The exact sample size (*n*) for each experimental group/condition, given as a discrete number and unit of measurement
☐	☒	A statement on whether measurements were taken from distinct samples or whether the same sample was measured repeatedly
☐	☒	The statistical test(s) used AND whether they are one- or two-sided<br>*Only common tests should be described solely by name; describe more complex techniques in the Methods section.*
☐	☒	A description of all covariates tested
☐	☒	A description of any assumptions or corrections, such as tests of normality and adjustment for multiple comparisons
☐	☒	A full description of the statistical parameters including central tendency (e.g. means) or other basic estimates (e.g. regression coefficient) AND variation (e.g. standard deviation) or associated estimates of uncertainty (e.g. confidence intervals)
☐	☒	For null hypothesis testing, the test statistic (e.g. *F*, *t*, *r*) with confidence intervals, effect sizes, degrees of freedom and *P* value noted<br>*Give P values as exact values whenever suitable.*
☒	☐	For Bayesian analysis, information on the choice of priors and Markov chain Monte Carlo settings
☒	☐	For hierarchical and complex designs, identification of the appropriate level for tests and full reporting of outcomes
☒	☐	Estimates of effect sizes (e.g. Cohen's *d*, Pearson's *r*), indicating how they were calculated

Our web collection on statistics for biologists contains articles on many of the points above.

Software and code

Policy information about availability of computer code

Data collection	Clampex 10.7, Clamfit 10.7
Data analysis	Origin 8.1.J, Prism 9

For manuscripts utilizing custom algorithms or software that are central to the research but not yet described in published literature, software must be made available to editors and reviewers. We strongly encourage code deposition in a community repository (e.g. GitHub). See the Nature Research guidelines for submitting code & software for further information.

Data

Policy information about availability of data

All manuscripts must include a data availability statement. This statement should provide the following information, where applicable:

- Accession codes, unique identifiers, or web links for publicly available datasets
- A list of figures that have associated raw data
- A description of any restrictions on data availability

Provide your data availability statement here.

April 2020

Field-specific reporting

Please select the one below that is the best fit for your research. If you are not sure, read the appropriate sections before making your selection.

☒ Life sciences ☐ Behavioural & social sciences ☐ Ecological, evolutionary & environmental sciences

For a reference copy of the document with all sections, see nature.com/documents/nr-reporting-summary-flat.pdf

Life sciences study design

All studies must disclose on these points even when the disclosure is negative.

Sample size	We repeated experiments with numbers more than 10, and these numbers are sufficient to make a conclusions.
Data exclusions	No data exclusions
Replication	Replications were successful.
Randomization	Not applicable
Blinding	Not blinded

Reporting for specific materials, systems and methods

We require information from authors about some types of materials, experimental systems and methods used in many studies. Here, indicate whether each material, system or method listed is relevant to your study. If you are not sure if a list item applies to your research, read the appropriate section before selecting a response.

Materials & experimental systems

n/a	Involved in the study	
☐	☒	Antibodies
☐	☒	Eukaryotic cell lines
☒	☐	Palaeontology and archaeology
☐	☒	Animals and other organisms
☒	☐	Human research participants
☒	☐	Clinical data
☒	☐	Dual use research of concern

Methods

n/a	Involved in the study	
☒	☐	ChIP-seq
☒	☐	Flow cytometry
☒	☐	MRI-based neuroimaging

Antibodies

Antibodies used	rabbit anti-ANO1 antibody (Abcam, ab53213, 1:5 for Western blotting), (Abcam, ab53212, 1:100 for immunoprecipitation); rabbit anti-phospho-ERK (extracellular signal-related kinase) antibody (Cell Signaling Technology, #4370, 1:1000); rabbit anti-phospho-p38 antibody (Cell Signaling Technology, #4511, 1:1000); rabbit anti-phospho-JNK (c-Jun N-terminal Kinase) antibody (Cell Signaling Technology, #4668, 1:1000); rabbit anti-ERK antibody (Cell Signaling Technology, #4695, 1:1000); abbit anti-p38 antibody (Cell Signaling Technology, #8690, 1:1000); rabbit anti-JNK antibody (Cell Signaling Technology, #9252, 1:1000); mouse anti-β-actin antibody (Abcam, ab6276, 1:2500); rabbit anti-TRPV3 antibody (Cell Signaling Technology, #3484, 1:1000 for Western blotting; 1:50 for immunoprecipitation); anti-rabbit-HRP antibody (Cell Signaling Technology, #7074, 1:2000); anti-mouse-HRP antibody (Cell Signaling Technology, #7076, 1:2000)
Validation	Quality if all the antibodies was validated with each protein produced in HEK293T cells.

Eukaryotic cell lines

Policy information about cell lines

Cell line source(s)	HEK293T cells, NHEK
Authentication	ATCC CRT-3216, Krabo
Mycoplasma contamination	No contamination
Commonly misidentified lines (See ICLAC register)	*Name any commonly misidentified cell lines used in the study and provide a rationale for their use.*

Animals and other organisms

Policy information about studies involving animals; ARRIVE guidelines recommended for reporting animal research

Laboratory animals	7-weeks-old C57BL/6NCr male mice were used. Mice were kept under a 12-hour light-dark cycle, at 24 °C with unlimited food and water. TRPV3-deficient (TRPV3KO) mice were maintained on a C57BL6/NCr background.
Wild animals	Not applicable.
Field-collected samples	Not applicable.
Ethics oversight	All animal care and experimental procedures were approved by the institutional Animal Care and Use Committee of the National Institute of Natural Sciences, and performed according to the National Institutes of Health and National Institute for Physiological Sciences guidelines.

Note that full information on the approval of the study protocol must also be provided in the manuscript.
