# Supplementary figures and images for: TRPV3-ANO1 interaction positively regulates wound healing in keratinocytes
Source: Commun Biol. 2023 Jan 23;6:88. doi: 10.1038/s42003-023-04482-1 (PMC9870996; doi:10.1038/s42003-023-04482-1)

control

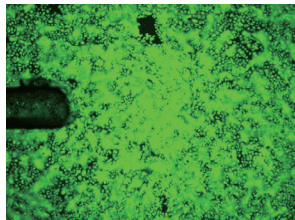

ANO1 Inhibitor

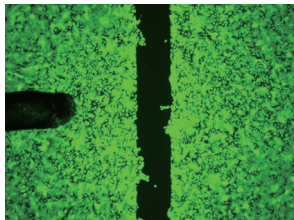

Supplement: Supplementary file 6 — Features Image [file 42003_2023_4482_MOESM6_ESM.pdf]
